# Clinical and Anatomical Characteristics of Perforator Aneurysms of the Posterior Cerebral Artery: A Single-Center Experience

**DOI:** 10.3390/brainsci14090934

**Published:** 2024-09-19

**Authors:** Anahita Malvea, Shigeta Miyake, Ronit Agid, Hugo Andrade Barazarte, Richard Farb, Timo Krings, Pascal John Roger Mosimann, Patrick Joseph Nicholson, Ivan Radovanovic, Karel Terbrugge, Robert Willinsky, Joanna Danielle Schaafsma, Eef J. Hendriks

**Affiliations:** 1 Division of Neuroradiology, Joint Department of Medical Imaging, Toronto Western Hospital, University Health Network, 399 Bathurst St, Toronto, ON M5T 2S8, Canada; 2 Department of Neurosurgery, Yokohama City University School of Medicine, Yokohama 236-0027, Japan; 3 Department of Neurosurgery, Toronto Western Hospital, University Health Network, Toronto, ON M5G 2C4, Canada; 4 Department of Neuroradiology, Beaumont Hospital, BL6 4LA Dublin, Ireland; 5 Department of Neurology, University Health Network, Toronto, ON M5G 2C4, Canada

**Keywords:** cerebral aneurysm, posterior cerebral artery, perforator artery, endovascular coiling

## Abstract

Introduction: Posterior cerebral artery (PCA) aneurysms represent up to 1% of all cerebral aneurysms. P1-P2 perforator aneurysms are thought to be even less prevalent and often require complex treatment strategies due to their anatomical and morphological characteristics, with risk of a perforator infarct. We studied the treatment of P1-P2 perforator aneurysms in a single-center cohort from a high-volume tertiary center, reporting clinical and anatomical characteristics, treatment strategies, and outcomes. Methods: A retrospective analysis of adult patients with a P1-P2 perforator aneurysm who presented at our institution between January 2000 and January 2023 was performed. The patients were analyzed for demographics, clinical presentation, imaging findings, treatment techniques, outcomes, and complications. Subgroup analyses between ruptured versus non-ruptured cases were included. Results: Out of 2733 patients with a cerebral aneurysm, 14 patients (0.5%) presented with a P1-P2 perforator aneurysm. All six patients with a ruptured aneurysm were treated by endovascular coiling, of whom one patient (16.7%) required surgical clipping of a recurrence. One out of eight (12.5%) patients with unruptured aneurysms was treated by surgical clipping. P1-P2 perforator aneurysms predominantly affected middle-aged individuals (median 59.5 years), with 10/14 (71.4%) being female. Endovascular coiling was the primary treatment modality overall, yielding favorable technical outcomes, however, it was complicated by a perforator infarct in two patients (33.3%) without new permanent morbidity or mortality secondary to treatment. Conclusions: P1-P2 perforator aneurysms are a rare subtype of intracranial aneurysm. Endovascular coiling could present an effective treatment modality; however, care should be taken for ischemic complications in the dependent perforator territory. Larger studies are required to provide more insights.

## 1. Introduction

In general terms, posterior cerebral artery (PCA) aneurysms account for 1% of all intracranial aneurysms [1] and for 7% of aneurysms in the posterior circulation [2]. Up to 54% of patients present with a rupture [3]. Saccular aneurysms are found in 46% and 52% (ruptured versus unruptured), fusiform aneurysms in 15% and 12%, dissecting aneurysms in 21% and 14%, and serpentine aneurysms in 2% and 4%, respectively [1]. Given the high percentage of dissecting or fusiform aneurysms, endovascular treatment often involves stent-assisted coiling, flow diverter treatment [4], or parent vessel occlusion (PVO) [5], rather than unassisted coiling [6]. Essibayi et al., 2002 reported the treatment modalities of ruptured PCA aneurysms, with microsurgery being used in 35.8%, PVO in 34.9%, and reconstructive endovascular treatment in 24.3% [3]. There is an overall treatment-related complication rate of 15%, with hemorrhagic complications accounting for 3% [5] and ischemic complications occurring in approximately 12% of cases. Treatment-related complications were most commonly seen in the PVO group (38.1%) [3], most frequently leading to hemianopia.

**Figure 1:**
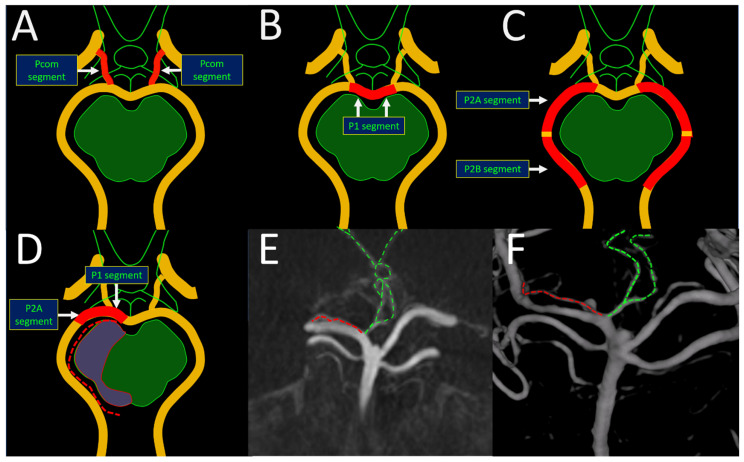
Axial schematic illustrations of the segments of PCA perforators at the level of the midbrain and optic chiasm (**A**–**C**) representing the 3 major thalamoperforating groups, i.e., Pcom, P1, and P2, supplying different thalamic territories. The tuberothalamic arteries originate from the middle third of the PcomA (**A**), supplying the ventral thalamus. The paramedian P1 perforators originate from the P1 or precommunicating segment (**B**) and supply the medial thalamus and/or rostral midbrain. The inferolateral perforators, also known as geniculate perforators or direct perforators, originate from the P2 or postcommunicating segment (**C**) and can be divided into the P2A and P2B segments, or crural cistern and ambient cistern segments. The P2A segment supplies the lateral thalamus, whereas the P2B classically supplies the inferolateral pulvinar. An anatomical variant and replacement of the paramedian P1 perforators is the artery of Percheron, representing a single dominant trunk with bilateral supply to the thalamus and/or midbrain ((**E**,**F**); green dotted lines) and originating of the P1 segment. An anatomical variant replacing the inferolateral P2 perforators is the collicular artery (also known as the quadrigeminal or circumcollicular artery), coursing medially to the main P2 segment around the cerebral peduncle towards the collicular plate ((**D**–**F**); red dotted lines) and originating from the P1 or P2A segment. Its name is derived from the area of supply. An identical origin of the collicular artery and the artery of Percheron is possible (**E**,**F**), in which the nomenclature is defined by the territory of supply. The example case demonstrates the presence of a right P1 perforator aneurysm at the origin of the collicular artery and the artery of Percheron.

From an embryological perspective, the PCA originates from the anterior circulation [7]; however, it is considered a functional artery of the posterior circulation in adults [8]. The PCA is divided into segments: the P1 segment extends from the basilar artery bifurcation to the posterior communicating artery confluence, the P2A segment runs ventrally to the midbrain, the P2B segment is located lateral to the midbrain, the P3 segment is located in the quadrigeminal cistern, and the P4 segment represents the cortical branches (Figure 1) [9]. Various perforating arteries arise from these segments, including the tuberothalamic arteries from the Pcom segment, the paramedian arteries from the P1 segment, and the inferolateral arteries from the P2 segment (Figure 1) [10,11], each with their characteristic supply to the thalamus and/or the midbrain.

The P1 and P2 segments can give rise to unique and clinically significant perforator arterial variation, namely the artery of Percheron and a dominant collicular artery [12]. The artery of Percheron is the anatomical variant artery replacing the normally present bilateral paramedian P1 perforators with a single dominant unilateral trunk originating of the P1 segment that subsequently divides, providing bilateral supply to the thalamus and/or midbrain. The collicular artery is the anatomical variant replacing the inferolateral P2 perforators, coursing medially and parallel to the main P2 segment around the cerebral peduncle, towards the collicular plate, and originating from the P1 or P2A segment (Figure 1).

The incidence of aneurysms of the artery of Percheron and collicular artery is unknown and treatment outcomes are limited to case reports [13,14,15,16]. Retrospective cohort studies on PCA aneurysms lack both demographics and outcomes of perforator aneurysms [2] or mainly focus on perforator aneurysms of the basilar artery [16,17,18]. In this study, we review the incidence, clinical presentation, and treatment outcomes of P1-P2 perforator aneurysms and further discuss their anatomical characteristics. Further insight into the clinical outcomes and detailed anatomy could improve pattern recognition in daily practice and the treatment of this rare and underreported entity.

## 2. Methods

Following institutional ethics review board approval, we conducted a retrospective analysis of a prospectively collected cerebral aneurysm database at our institution, a tertiary referral center.

The database containing a list of patients with intracranial aneurysms who presented between January 2000 and January 2023 was manually searched (A.M.) for patients meeting the following criteria. Patients were included in this study if they were: (1) over 17 years of age, (2) diagnosed with a perforator aneurysm of the P1 or P2 segment of the posterior cerebral artery (3) presented or were treated between 2000 and 2023, and (4) had at least cross-sectional CTA and/or MRA imaging available. The identification of P1 or P2 segment aneurysms was made by first excluding the patients whose reports did not include a posterior circulation aneurysm. Following that, the list was narrowed to include only posterior circulation aneurysms, and all patients described to have basilar tip aneurysms were excluded. All images in the shortlist were reviewed by A.M. and E.J.H. to identify patients specifically with a P1-P2 perforator aneurysm.

Data on age, sex, clinical presentation, past medical history (including hypertension, smoking, family history, multiplicity of aneurysms), Modified Rankin Scale (mRS), treatment techniques, complications, and clinical outcomes were collected through a chart review.

Statistical analyses were performed to compare the characteristics of ruptured versus non-ruptured subgroups (Table 1). The results are presented as means for continuous variables and frequency (percentage) for categorical data. Data were analyzed using the *t*-test for continuous variables and Fisher’s exact test to compare categorical data between groups. All *p*-values were two-sided, and a *p*-value of <0.05 was considered statistically significant. JMP Pro 15 (SASA Institute, Cary, NC, USA) was used for descriptive statistics.

## 3. Results

### 3.1. Patients

Between 2000 and 2023, 2733 patients with a cerebral aneurysm presented to our institution. Of those, 14 patients (0.5%) had a perforator aneurysm of the P1 or P2 segment of the posterior cerebral artery (Table 2). The median age of these 14 patients was 59.5 years (range 35–83) and 10 (71%) were female. Nine patients (69.2%) had a history of smoking and 8 (66.7%) had a history of hypertension (in five patients it was controlled with medication). None of the patients had a known family history of cerebral aneurysms. Six patients (43%) with a P1 or P2 segment perforator aneurysm had a single cerebral aneurysm, including one with an arteriovenous malformation (AVM), and eight patients presented with more than one cerebral aneurysm.

### 3.2. Treatment Modalities and Clinical Outcome

Six of the P1-P2 perforator aneurysm patients (43%) presented with a rupture, and eight patients (57%) had an unruptured P1-P2 perforator aneurysm. The average diameter of the P1-P2 perforator aneurysms was 3 mm (range 1.5–8.5). In our institution, unruptured posterior circulation aneurysms (including P1-P2 perforator aneurysms) are treated after a multidisciplinary discussion of each individual case, reviewing the size (generally at least 4–5 mm to consider treatment), risk factors (hypertension, smoking, family history, previous SAH, PHACES score), age, and comorbidities. Nowadays, endovascular therapy is the first choice for basilar tip and proximal PCA aneurysms, provided it is technically safe and feasible, with stable arterial access. All six ruptured P1-P2 perforator aneurysms were treated with endovascular coiling, with one requiring surgical clipping of a recurrence during follow-up. Following the multidisciplinary discussion, one unruptured wide-necked P1-P2 aneurysm (patient no 6) was treated with uncomplicated surgical clipping, given its size (4 × 3.5 mm). The remainder of the unruptured aneurysms were treated conservatively. Out of the six acute endovascular coiling cases, two patients suffered from silent perforator infarcts (Table 2: patient no 5 and 9). Patient no 5 had a past medical history of astrocytoma resection, radiation, and hydrocephalus treated with VP-drains and demonstrated an asymptomatic small acute infarct in the left ventral thalamus on day 4 MRI of the brain after endovascular coiling. Patient no 9 presented with a subarachnoid hemorrhage and mild left-sided drift, remaining stable after endovascular coiling, with an acute infarct in the right ventral thalamus on day 10 MRI of the brain. The patient suffered an acute respiratory decompensation due to pulmonary edema on postprocedural day 2 and cardiac arrest on day 4 and was discharged to a long-term care facility on day 90. All patients treated within the last 10 years (patient no. 9, 13, 14) had an immediate post-treatment mRS of 0. Patient no. 9 suffered from a respiratory-related cardiac arrest during hospital admission. Patient no. 10, without aneurysm closure treatment, was wheelchair-bound secondary to a prior moyamoya-related hemorrhage, and the remaining six patients were asymptomatic.

## 4. Discussion

The main findings of our study are: (1) P1-P2 perforator aneurysms are rare, with an incidence of 0.5% in a large single-center cerebral aneurysm cohort, (2) the technical results of endovascular coiling were good, with one case requiring retreatment, (3) endovascular coiling resulted in perforator infarcts in two patients; however, without new permanent morbidity secondary to treatment.

Compared to perforator aneurysms of the proximal anterior cerebral artery (A1 segment) [19,20], the horizontal segment of the middle cerebral artery (M1 segment) [21] and the basilar artery [17], P1-P2 perforator aneurysms have been less frequently reported on. Aneurysms of the A1 segment account for approximately 1% of all cerebral aneurysms and are associated with treatment-related complications exceeding 15% [19,20]. M1 aneurysms are associated with postprocedural infarcts in up to 22% of cases, a mortality rate of approximately 6% [21], and are often associated with hypertension, moyamoya disease, and AVMs. Chau et al. reported treatment-related complications in 17% of basilar artery perforator aneurysms [22]. As such, one may conclude that perforator aneurysms in any given location are associated with a moderate to high risk of ischemic complications due to treatment.

**Figure 2:**
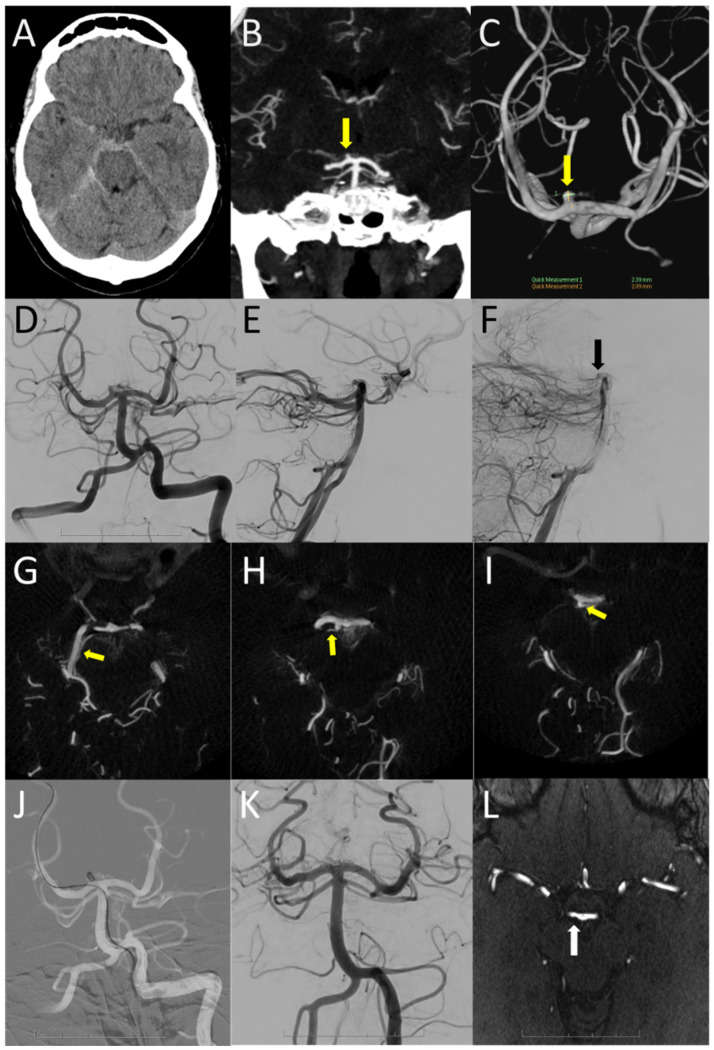
Case no 13 involves a 64-year-old female patient with a subarachnoid hemorrhage (GCS 15, WFNS 1) with the epicenter in the right interpeduncular cistern (**A**), with suspicion of a small superiorly and posteriorly pointing right P1 aneurysm ((**B**,**C**); yellow arrow). AP (**D**) and lateral left vertebral artery injections (**E**,**F**) confirm a right P1 aneurysm, with stases of contrast in the dome in the late arterial phase ((**F**); black arrow). Axial Xpert CT angiography reconstructions demonstrate the course of the right collicular artery in the crural and ambient cisterns along the posterior cerebral artery ((**G**–**I**); yellow arrows) originating medially from the dome of the aneurysm ((**I**); yellow arrow). An unassisted coiling with balloon presence was performed with two coils (**J**) resulting in direct (**K**) and one-year follow-up closure of the aneurysm on MRA time-of-flight (**L**). There is subtle indentation of the coil mass in the parent artery ((**L**); white arrow).

From a neuroanatomical point of view, recognizing the relationship with a perforator artery is important to reduce the complication risks in P1-P2 perforator aneurysm treatment. Features of this subtype of aneurysm are: (1) its off-midline position in relation to the basilar tip (2) its posterior or posterior medial angulation of the aneurysm dome in relation to the P1 or P2 segment, respectively, (3) the origin of a (dominant) perforator trunk juxtaposed to or at the neck of the aneurysm, as opposed to an infundibulum with a perforator at the tip of the conus, (4) stasis of contrast in the aneurysm dome observed in ruptured cases on angiography (Figure 2). Special consideration should be given to the presence of tuberothalamic arteries, a dominant paramedian artery (the artery of Percheron), and the collicular artery (Figure 1) [12]. The tuburothalamic arteries (TTAs) originate from the middle third of the PcomA and classically supply the ventral thalamus. The paramedian P1 arteries supply the medial thalamus and/or rostral midbrain and may supply the anterior thalamus when the TTA is hypoplastic. The inferolateral perforators or geniculate P2 perforators supply the lateral thalamus and inferolateral pulvinar. Both the artery of Percheron and the collicular artery can have an identical origin, in which the nomenclature is defined by the supplied territory (Figure 2). Thus, the collicular artery may originate from the P1 segment, with perforators branching extensively from the mesencephalon to the midbrain, varying among individuals [23].

Limited case reports on P1-P2 perforator artery aneurysm treatment exist (Table 3). Sparacia et al., 2018 described a ruptured aneurysm of the artery of Percheron treated with flow diversion resulting in closure of the aneurysm and occlusion of the artery of Percheron, with secondary bilateral paramedian thalamic infarcts [14]. Oishi et al., 2016 reported on two large P1 aneurysms in close relationship to the artery of Percheron, treated with parent vessel occlusion, resulting in extensive paramedian artery occlusion in one secondary to occlusion of the artery of Percheron [13]. Given the results of the present study and prior case reports, endovascular treatment with (balloon-assisted) coiling, if technically feasible, could represent a viable and preferred treatment of P1-P2 perforator aneurysms. The treatment of a perforator aneurysm inherently poses a risk of a perforator infarct. Risk reduction measures could be an undercoiling technique if technically feasible (i.e., selectively coiling the dome and rupture point and undercoiling of the neck and origin of the perforator), coiling under full heparinization, and/or starting low dose aspirin following endovascular treatment. Surgical clipping is challenging, given the presence of a perforating artery adherent to the neck of the aneurysm [24,25]. Furthermore, clipping in this region poses challenges due to the deep location near cranial nerves, increasing the risk of complications in a restricted surgical field [26,27]. Consequently, treatment strategies differ from those employed for M1 aneurysms [21]. Moreover, trapping and bypass procedures, commonly used for more distal PCA aneurysms, are not suitable. While FD has been considered beneficial [4], reports of FD for posterior circulation indicate a risk of perforator vessel infarction of approximately 7%, along with a high mortality rate [8], raising safety concerns, particularly in the acute phase of a subarachnoid hemorrhage.

Limitations of our study are associated to the retrospective nature of this single-center series with insufficient case numbers for proper statistics. Furthermore, due to the long inclusion period of this study, we were unable to obtain comprehensive clinical data on each patient. Lastly, the primary treatment strategy of basilar tip and P1-P2 aneurysms has shifted since the nineties from primary surgical clipping toward a primary endovascular treatment strategy due to the deep location in the brain with a restricted surgical field, as previously mentioned, as well as the evolution of endovascular therapy, with improved access and materials, such as microcatheters and microcoils. The number of coiling versus clipping patients is unequally distributed in our study, and the cases with perforator infarcts were treated in 2007 and 2014. Therefore, it is difficult to postulate whether a shift in treatment strategy and/or improvements in technology have had an influence.

To the best of our knowledge, this is the first retrospective cohort study on patients with a ruptured or unruptured P1-P2 perforator aneurysm addressing clinical outcomes, technical aspects of treatment modalities, and further pointing out anatomical details that could improve pattern recognition and the treatment of this rare entity.

## 5. Conclusions

P1-P2 perforator aneurysms are rare and require careful management due to their propensity for ischemic complications. Endovascular coiling could present an effective treatment modality; however, care should be taken for ischemic complications in the dependent perforator territory. Larger studies are required to provide deeper insight.

## Figures and Tables

**Table 1:** Subgroup analyses of P1-P2 perforator aneurysms.

P1-P2 Perforator Aneurysm	Total No	Treatment Group	Conservative Group	*p*-Value
No of cases	14	7 (50%)	7 (50%)	-
Age	59.5 (35–83)	61 (50–76)	54 (35–83)	0.3
Sex (female)	10 (71.4%)	5 (71.4%)	5 (71.4%)	>0.999
Smoking (current and past)	9 (69.2%)	4 (66.7%)	5 (71.4%)	0.66
Hypertension	8 (66.7%)	5 (83.3%)	3 (50%)	0.55
>1 cerebral aneurysm	8 (57.1%)	2 (28.6%)	6 (85.7%)	0.1
Ruptured	6 (42.9%)	6 (85.7%)	0 (0%)	<0.01
Maximum diameter (mm)	3 (1.5–8.5)	3.6 (1.5–8.5)	2.8 (2–3)	0.04
Outcomes				
Remnant after treatment	-	3 (42.9%)	NA	-
Permanent morbidity after treatment	-	0 (0%)	NA	-
Complications after treatment	-	2 (33.3%)	NA	

**Table 2:** Characteristics of patients with a P1-P2 perforator aneurysm and treatment outcomes.

Patient	Ruptured/ Unruptured	Year of Procedure or Presentation	Initial Presentation	Baseline mRS	Post Treatment-mRS	Total AA	Hypertension	Past History Smoking	Treatment Complications	Permanent Morbidity	Treatment of P1-P2 Aneurysm	Remnant	Treatment of Other Aneurysms
1	Unruptured	2003	Asymptomatic	0	NA	1	No	No	NA	None	None	NA	NA
2	Ruptured	2005	Symptomatic	0	0	1	Yes	Yes	No	None	Coiling	Yes *	NA
3	Unruptured (PCom Ruptured)	2005	Asymptomatic (Pcom symptomatic)	0	NA	3	Unknown	Yes	NA	None	None	NA	Coiling Pcom AA
4	Ruptured	2004	Symptomatic	Unknown	Unknown	1 AVM	Unknown	Unknown	Unknown	Unknown	Coiling	Yes *	NA
5	Ruptured	2007	Symptomatic			1	Yes–Tx	Yes	Acute perforator infarct in caudothalamic groove post coiling	Due to history of resected astrocytoma, radiation, hydrocephalus	Coiling followed by clipping	Yes	NA
6	Unruptured	2008	Asymptomatic	0	NA	3	Yes–Tx	Yes	NA	None	Clipping	No	Coiling basilar AA
7	Unruptured	2003	Asymptomatic	0	NA	3	No	Yes	NA	None	None	NA	Clipping MCA and anterior choroidal AA
8	Unruptured	2008	Asymptomatic	0	NA	3	Yes	Yes	NA	None	None	NA	Coiling ICA terminus AA
9	Ruptured	2014	Symptomatic	0	0	4	Yes–Tx	No	Acute infarct right thalamus	Due to cardiac arrest	Coiling	No	Coiling Pcom AA
10	Unruptured	2014	Asymptomatic	5	NA	2	No	No	NA	Due to MoyaMoya related ICH	None	NA	NA
11	Unruptured	2002	Asymptomatic	0	NA	2	Yes–Tx	Yes	NA	No	None	NA	NA
12	Unruptured (Ruptured ACA)	1997	Asymptomatic (Symptomatic ACA)	0	NA	3	Yes–Tx	Yes	NA	None	None	NA	Clipping ACA AA
13	Ruptured	2023	Symptomatic	0	0	1	No	No	No	None	Coiling	No	NA
14	Ruptured	2022	Symptomatic	0	0	1	Yes	Yes	No	None	Coiling	No	NA

* Remnant without indication for retreatment. Abbreviations: AA arterial aneurysm; NA not applicable; Tx treatment.

**Table 3:** Literature on P1-P2 perforator aneurysms.

Author	Year	No. of Patients	Age	Aneurysm Description	Treatment	Technique	Complication	Clinical Outcome
Da Ros et al. [16]	2020	1	Unknown	P1 aneurysm	Endovascular	Flow diversion	None	mRS 0
Sparacia et al. [14]	2018	1	48	Artery of Percheron aneurysm	Endovascular	Flow diversion	Bilateral paramedian thalamic infarcts	Hydrocephalus Discharge to rehab
Giordan et al. [15]	2018	1	47	Pseudoaneurysm P1 perforator	DSA only	NA	None	Bilateral third nerve palsy
Oishi et al. [13].	2016	2	49 54	P1-P2 aneurysm P1 aneurysm	Endovascular Endovascular	Parent vessel occlusion Parent vessel occlusion	None Extensive thalamic infarction	Unchanged Decline post treatment to MRS 3 (pretreatment MRS 0)

## Data Availability

The authors confirm that the data supporting the findings of this study are available within the article.

## Abbreviations

Posterior cerebral artery (PCA), parent vessel occlusion (PVO), reconstructive endovascular treatment (rEVT), arteriovenous malformation (AVM), posterior communicating artery (PcomA), flow diversion (FD), tuberothalamic artery (TTA)
